# 

**Published:** 1969-10

**Authors:** 


### *Contents*

| | page |
|---|---|
| CHRONIC RHEUMATIC ENDOCARDITIS. *J. Goodwin* ... | 107 |
| SURGICAL ASPECTS OF RHEUMATIC HEART DISEASE. *T. H. Sellors* ... ... ... ... ... ... ... | 115 |
| CARDIAC LESIONS IN CHILDREN WITH "HYPERCALCAEMIC" FACIES. *S. C. Jordan* ... ... ... ... | 121 |
| TWINS WITH RHEUMATIC DISEASES. *A. St. J. Dixon* ... | 125 |
| PHARMACOLOGICAL FIBRINOLYSIS. *G. R. Fearnley* ... | 131 |
| ARTHRITIS AND THE HEART. *J. A. Cosh* ... ... ... | 145 |
| CLAUDICATION. *D. N. Walder* ... ... ... ... ... | 153 |
| LIPOPROTEIN DEFICIENCY DISORDERS. *J. K. Lloyd* ... | 159 |
| FINGER-PRINTING IN CONGENITAL HEART DISEASE. *T. J. David* ... ... ... ... ... ... ... | 167 |
| HOME CARE OF MYOCARDIAL INFARCTION. *H. G. Mather* ... ... ... ... ... ... ... ... | 171 |
| THE CARE OF PEPTIC ULCER PATIENTS. *N. C. Tanner* ... | 179 |
| AORTIC VALVE REPLACEMENT. *R. G. Charles* and *C. M. Morgans* ... ... ... ... ... ... ... | 185 |
| EVALUATION OF CLINICAL PHENOMENA IN PSYCHIATRY. *J. C. Little* ... ... ... ... ... ... | 191 |
| DRUGS AND LIVER DISEASE. *A. E. Read* ... ... ... | 199 |
| PSEUDO-OBSTRUCTION OF THE SMALL BOWEL. *F. Nahai* | 209 |
| ATRIAL BRADYCARDIA. *D. B. Shaw* and *D. Eraut* ... ... | 213 |

